# Supplementary material for: Deletion of wfs1 Impairs Oligodendrocyte Precursor Cells Dorsal Distribution and Myelination Through the wfs1-hmgcs1 Axis in Zebrafish
Source: Biology (Basel). 2026 Aug 16;15(16):1402. doi: 10.3390/biology15161402 (PMC13510151; doi:10.3390/biology15161402)
Supplement: Supplementary file 1 [file biology-15-01402-s001.zip › File S1-Transcriptome sequencing data and differentially expressed genes (DEGs) in wfs1-- zebrafish at 5 dpf/2.KEGG/wfs1KOvsWT/UP/src/dre00380.html]

dre00380


- dre:571967

- Up regulated genes

571967(1.93014769457854)

- dre:571967

- Up regulated genes

571967(1.93014769457854)

- dre:571967

- Up regulated genes

571967(1.93014769457854)

- dre:571967

- Up regulated genes

571967(1.93014769457854)

- dre:393860

- Up regulated genes

393860(1.20497977076277)

- dre:447801

- Up regulated genes

447801\_1(8.60129050664356)

- dre:103911233

- Up regulated genes

103911233(1.84859647244981)
- dre:571201

- Up regulated genes

103911241(5.46067952567297)

- dre:103911233

- Up regulated genes

103911233(1.84859647244981)
- dre:571201

- Up regulated genes

103911241(5.46067952567297)

- dre:368239

- Up regulated genes

368239(1.14034759711527)

- dre:368239

- Up regulated genes

368239(1.14034759711527)

- dre:555401

- Up regulated genes

555401(1.88844791660122)

Close
